# Construction of a ferroptosis scoring system and identification of LINC01572 as a novel ferroptosis suppressor in lung adenocarcinoma

**DOI:** 10.3389/fphar.2022.1098136

**Published:** 2023-01-04

**Authors:** Lingling Hong, Xuehai Wang, Weiming Cui, Fengxu Wang, Weiwei Shi, Shali Yu, Yonghua Luo, Lixin Zhong, Xinyuan Zhao

**Affiliations:** ^1^ Nantong Hospital of Traditional Chinese Medicine, Affiliated Traditional Chinese Medicine Hospital of Nantong University, Nantong, China; ^2^ Department of Occupational Medicine and Environmental Toxicology, Nantong Key Laboratory of Environmental Toxicology, School of Public Health, Nantong University, Nantong, China; ^3^ Department of Thoracic and Cardiac Surgery, Nanjing Brain Hospital, Nanjing, China; ^4^ Nantong Fourth People’s Hospital, Nantong, China; ^5^ Jiangsu Provincial Center for Disease Control and Prevention, Nanjing, China

**Keywords:** lung adenocarcinoma, long non-coding RNA, ferroptosis, N6-methyladenosine (m6A) methylation, signature, LINC01572

## Abstract

**Background:** Ferroptosis is a novel process of programmed cell death driven by excessive lipid peroxidation that is associated with the development of lung adenocarcinoma. N6-methyladenosine (m6a) modification of multiple genes is involved in regulating the ferroptosis process, while the predictive value of N6-methyladenosine- and ferroptosis-associated lncRNA (FMRlncRNA) in the prognosis of patients remains with LUAD remains unknown.

**Methods:** Unsupervised cluster algorithm was applied to generate subcluster in LUAD according to ferroptosis-associated lncRNA. Stepwise Cox analysis and LASSO algorithm were applied to develop a prognostic model. Cellular location was detected by single-cell analysis. Also, we conducted Gene set enrichment analysis (GSEA) enrichment, immune microenvironment and drug sensitivity analysis. In addition, the expression and function of the LINC01572 were investigated by several *in vitro* experiments including qRT-PCR, cell viability assays and ferroptosis assays.

**Results:** A novel ferroptosis-associated lncRNAs-based molecular subtype containing two subclusters were determined in LUAD. Then, we successfully created a risk model according to five ferroptosis-associated lncRNAs (LINC00472, MBNL1-AS1, LINC01572, ZFPM2-AS1, and TMPO-AS1). Our nominated model had good stability and predictive function. The expression patterns of five ferroptosis-associated lncRNAs were confirmed by polymerase chain reaction (PCR) in LUAD cell lines. Knockdown of LINC01572 significantly inhibited cell viability and induced ferroptosis in LUAD cell lines.

**Conclusion:** Our data provided a risk score system based on ferroptosis-associated lncRNAs with prognostic value in LUAD. Moreover, LINC01572 may serve as a novel ferroptosis suppressor in LUAD.

## Introduction

Lung cancer (LC) is the leading cause of cancer-related deaths globally (Bray et al., 2018). The number of patients with LUAD accounts for about 50% of all LC cases (Coudray et al., 2018; Chen et al., 2021). Most patients re at an advanced stage at the time of diagnosis, with dismal survival outcome. (Denisenko et al., 2018). LUAD is treated by surgery, radiotherapy and targeted therapy. Targeted therapy with its precise and efficient characteristics has played central part in clinical treatment (Imielinski et al., 2012). Therefore, it is crucial to find new therapeutic targets to improve the treatment of LUAD.

Long non-coding RNAs (lncRNAs) are transcripts of more than 200 nucleotides, and according to current reports, lncRNAs have no direct transcriptional ability to encode proteins (Peng et al., 2017; Geng et al., 2022). However, essential effects of lncRNAs in regulating RNA transcription, translation and protein dynamics have now been identified, and there is increasing evidence that lncRNAs have different roles in the pathogenesis of cancer (Ma et al., 2018; Song et al., 2021a). Researchers have recently used lncRNA microarrays, lncRNA sequencing, and qRT-PCR to identify lncRNAs that are differentially expressed in tumor tissues. Long non-coding RNA MALAT1 was upregulated in gastric carcinoma and positively regulates autophagy in multiple cancers (Wang et al., 2021; Gu et al., 2022). The levels of HOTAIR in metastatic breast cancer tissues were higher than normal breast epithelium and primary breast cancer foci, and high HOTAIR expression was associated with poorer prognosis in patients and with metastasis in the course of the disease (Gupta et al., 2010; Liu et al., 2022a).

N6-methyladenosine (m6A) is the most abundant epigenetic modification in eukaryotic mRNA and non-coding RNA, and this chemical modification process is dynamic and reversible (Ma et al., 2019; Liu et al., 2022b). It is generally accepted that m6A modifications are regulated by three proteins, including “writers,” “erasers,” and “readers” (He et al., 2019). LncRNA-PACERR, a crucial regulator of TAMs in the PDAC microenvironment, could enhance the expression of KLF12 in an m6A-dependent manner, thereby promoting cell viability and metastasis (Liu et al., 2022c). FTO mediates the m6A modification of LINC00022 and boost ubiquitination-mediated degradation of p21 to promote tumor growth in ESCC *in vivo* (Cui et al., 2021).

Ferroptosis, a subtype of programmed cell death, can be regulated through m6A methylation to maintain cell cycle and tissue homeostasis (Song et al., 2021b; Shen et al., 2021). Ferroptosis is mainly characterized by iron accretion and lipid peroxidation (Mou et al., 2019). Ma et al. (2021) found that m6A reader YTHDC2 can inhibit LUAD tumorigenesis by SLC7A11-dependent antioxidant function (Liu et al., 2021). In gastric cancer, lncRNA CBSLR interacts with YTHDF2, which reduces the expression of CBS mRNA, contributing to iron death resistance (Yang et al., 2022). In this study, we identified a group of specific lncRNAs associated explicitly with the prognostic status of LUAD. Moreover, these lncRNAs can further evaluate the guiding value of immune efficacy, immune infiltration, drug sensitivity, and biological function for clinical treatment.

## Materials and methods

### Data collection

The transcriptome data of LUAD cases were obtained from TCGA (https://portal.gdc.cancer.gov/). Patients with missing survival information were excluded. The FRGs were downloaded from FerrDb (http://www.zhounan.org/ferrdb/current/). In addition, we extracted the gene set for m6A regulators from previous literature (Du et al., 2021).

### Determination of ferroptosis and m6A related lncRNA (FMRlncRNA)

Pearson correlation analysis was used to screened out lncRNAs related to PRGs or m6A regulators The association was considered significant if the correlation coefficient |*R* ^2^| > .4 at *p* < .001. Differentially expressed lncRNAs (DElncRNAs) were selected by the “limma” package (Ritchie et al., 2015).

### Unsupervised gene clustering

Consensus clustering was applied with “ConsensusClusterPlus” package (Wilkerson and Hayes, 2010). To identify the favorable cluster value, the Delta area and cumulative distribution function (CDF) were estimated. Then we compared the clinical outcomes among subtypes using survival analysis.

### Development of FMRlncRNA model

We randomly classified the included cases (*n* = 500) into training and validation cohorts at a 1:1 ratio. The model was generated by stepwise Cox regression and LASSO algorithm. The risk score of each case with LUAD was evaluated according to the following formula: $\overset{n}{\underset{i=1}{\sum }}coeff_{i}*expression\;level\;of\;lncRNA_{i}$ , where coef is the coefficient of the model generated by Cox analyses.

All patients were classified by the median risk score into high- and low-risk groups.

### Survival analysis

The discrepancies in clinical outcome between groups were examined by Kaplan-Meier survival analysis. The reliability of model in outcome assessment was investigated by drawing ROC curves. The independent value of model in LUAD was verified *via* Cox relevant analyses.

### Single-cell analysis

The single-cell data set GSE123904 of LUAD was collected from the GEO database. We applied “Seurat” package to conduct data quality control and integration (Mangiola et al., 2021). The PCA analysis and t-SNE algorithm were utilized to determine cell subclusters. Using “FindAllMarkers” to obtain the specific biomarker of different cell population.

### Gene set enrichment analysis (GSEA)

We chose the Hallmark and KEGG as the reference gene sets. Then 1000 enrichment analyses were done with the default weighted method. Any gene set with FDR < .25 and *p* < .05 was regarded as significant.

### Immune activity analysis

Five bioinformatics algorithms (CIBERSORT, ESTIMATE, MCPcounter, ssGSEA, and TIMER) were applied to detect immune responses between two group. Additionally, we employed ssGSEA to evaluate the immunocyte infiltrating as well as immune function between two groups.

### Drug sensitivity analysis

The effect of chemotherapy was evaluated by Genomics of Drug Sensitivity in Cancer (GDSC8). The half-maximal inhibitory concentration (IC50) was estimated which represented the drug response.

### Cell culture and transfection

The LUAD cell lines (A549 and NCI-H2009) and bronchial epithelioid cells (HBE) were obtained from shanghai. The LUAD cells were cultured in RPMI-1640 medium and maintained in a humidified incubator at 37°C in 5% CO_2_. The silencing RNA against LINC01572 (si-LINC01572) were synthesized and purchased from RIBBIO (Guangzhou, China). The sequences of si-LINC01572 were shown as Supplementary Table S1. Lipofectamine 3000 (Invitrogen) was used to transfect siRNA and its negative control.

### CCK8 assay

5,000 cells per well were seeded in a 96-well plate to measure cell viability. Each well was replaced with fresh DMEM containing 10 µl of Cell Counting Kit-8 (CCK8) reagent. After 4 h of incubation at 37°C, the absorbance of each well was measured at 450 nm.

### EdU assay

We utilized Ribobio’s Edu staining kit to assess cell proliferation. 5000 cells were seeded in a 96-well plate. EdU solution (25 μM) was added to the well plate for 2 h the next day. Afterward, cells were fixed in 4% paraformaldehyde for 30 min, followed by 50 μl, 2 mg/ml glycine for 5 min. After incubation with 100 μl .5% Triton X-100, cells were incubated with 100 μl 1× Apollo^®^ 567 fluorescent staining solution for 30 min in a dark environment. The nuclei of the cells were stained with DAPI. Finally, the images were observed with an inverted fluorescence microscope.

### Reverse transcription-polymerase chain reaction (PCR)

Total RNA was extracted from cells using Trizol reagent according to the manufacturer’s instructions. RNA was then reverse-transcribed to cDNA with Primer-script Master Mix (Takara Bio, RR0236-1, Kusatsu, Japan). Quantitative PCR was performed with SYBR Green I Master Mix (Takara Bio, Q34-02, Kusatsu, Japan). Supplementary Table S1 displays primer sequences of all genes. The 2^−ΔΔ^Ct method was adopted for calculating relative gene expression, with GAPDH being the endogenous control.

### Determination of lipid peroxidation and iron content

Lipid peroxidation detection kits (Abcam) were used to evaluate the concentrations of the lipid peroxidation products MDA and 4-HNE. To investigate the degree of iron deposit, an iron assay kit (Abcam) was used for detection in cell lysates according to the manufacturer’s instructions. The results were measured using a microplate reader.

### Statistical analysis

All statistical data were analyzed using GraphPad 9.4 and the R software version 4.0.

## Results

### Ferroptosis and m6A-related lncRNA (FMRlncRNA) identification and unsupervised cluster analysis

**FIGURE 1:**
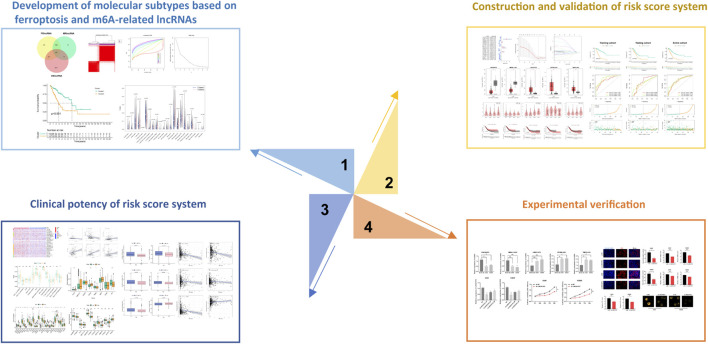
Flow chart.

The workflow of our research is shown in Figure 1.

**FIGURE 2:**
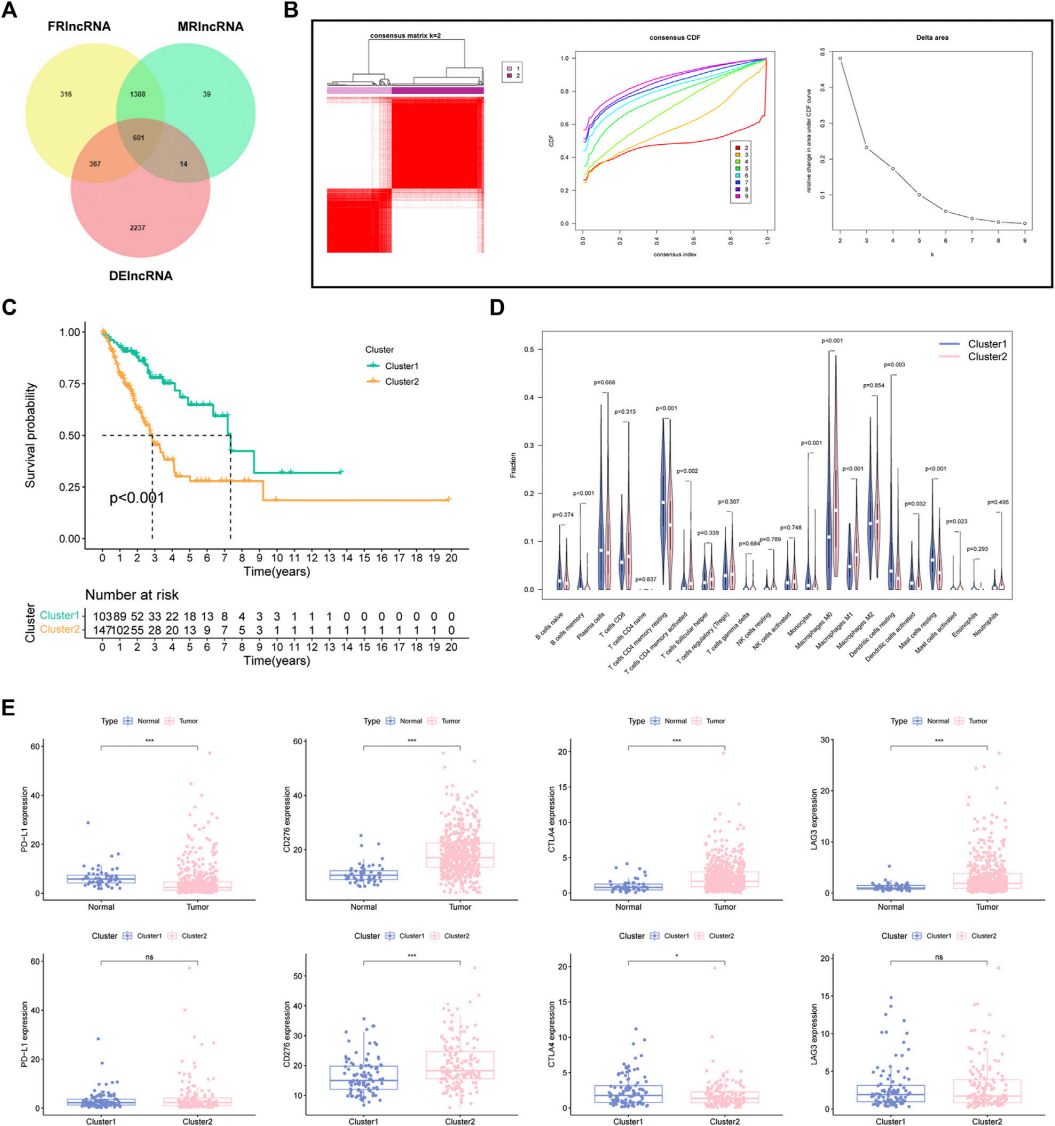
Determination of FMRlncRNA molecular subtype in LUAD **(A)** The Venn plot of intersection DElncRNAs. **(B)** Consensus clustering results. **(C)** Kaplan–Meier survival analysis, **(D)** immune cell differential analysis for patients between two subclusters. **(E)** Immune checkpoint analysis (ns > .05, *p < .05, ***p < .001).

Firstly, a total of 3219 DElncRNA were determined between LUAD specimens and normal control. Then, we screened 601 intersecting DEFMRlncRNAs for the next analysis (Figure 2A). Based on the 601 DEFMRlncRNAs, unsupervised cluster analysis suggested the ideal value of subcluster was two (Figure 2B). Survival analysis indicated that cluster1 had a notably better survival outcome than cluster2 (Figure 2C). Besides, there were also large differences in immunocytes between the two clusters (Figure 2D). We also tested whether the four immune checkpoints (PD-L1, CD276, CTLA4, LAG3), which differ in tumor and normal tissues, differed in two clusters, showing that CD276 expression was higher in cluster2 than in cluster1, while CTLA4 expression was lower than in cluster1. PD-L1 and LAG3 showed no significant difference in expression between the two clusters (Figure 2E).

### Establishment of FMRlncRNA model (FMRLM)

**FIGURE 3:**
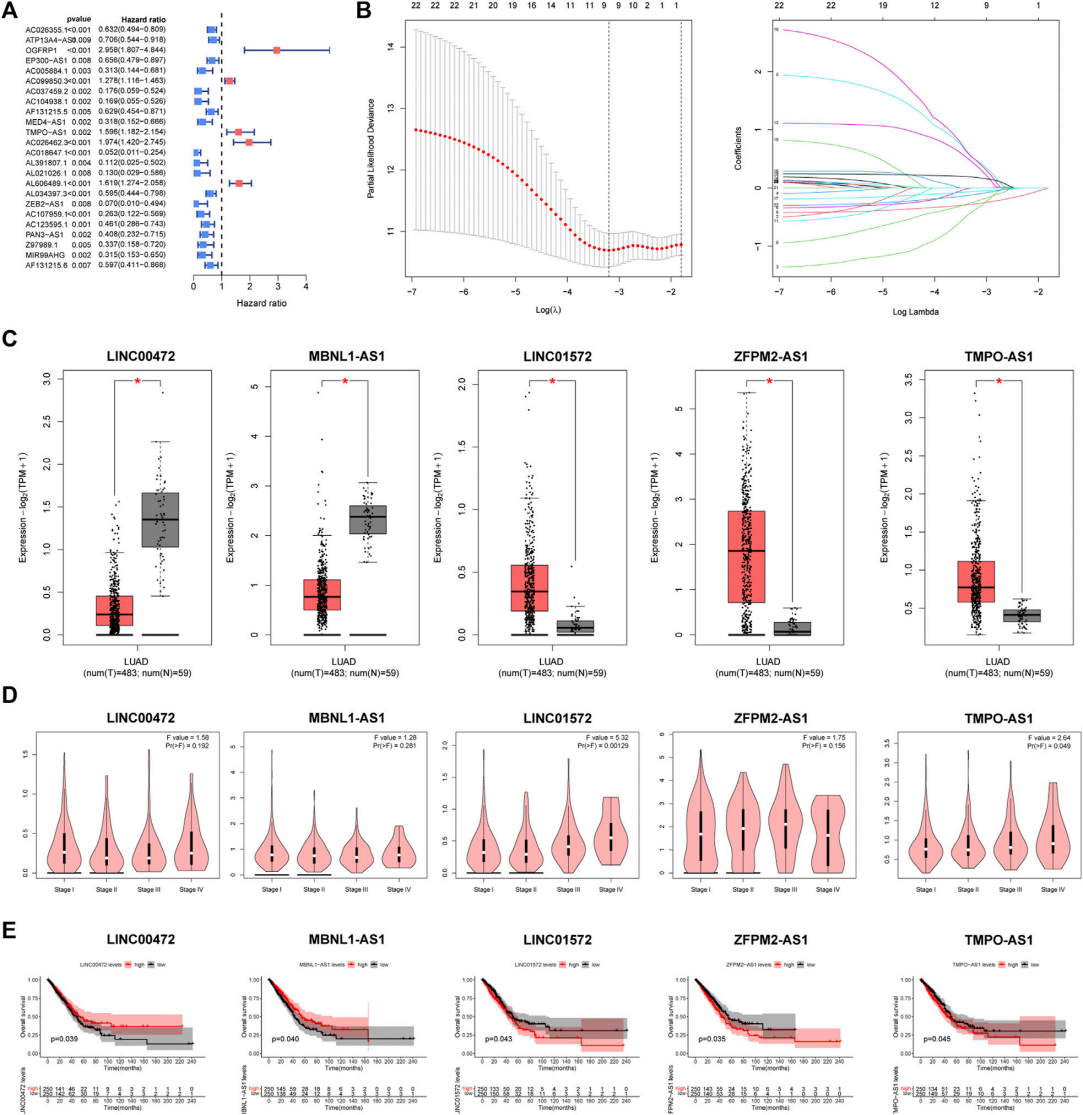
Creation process of the FMRLM **(A)** Univariate Cox regression analysis. **(B)** LASSO regression for avoiding overfit of the signature. **(C)** Analysis of differential expression of 5 lncRNAs in tumor and normal tissues, **(D)** relationship with patient tumor stage, and **(E)** impact on survival prognosis (*p < .05).

All LUAD samples were equally divided into the training (*n* = 250) and validation groups (*n* = 250). In the training group, based on univariate Cox, we found 37 FMRlncRNAs associated with survival outcome on the basis of univariate Cox (Figure 3A). To avoid over-fitting prognostic features, we performed LASSO regression (Figure 3B). Finally, a risk score system containing (LINC00472, MBNL1-AS1, LINC01572, ZFPM2-AS1, and TMPO-AS1) was generated by multivariate analysis. The risk model equation was: (1.754 × LINC01572) + (2.131 × TMPO-AS1) + (1.142 × ZFPM2-AS1) + (−2.366 × LINC00472) + (−1.351 × MBNL1-AS1). According to GEPIA2 portal, we found that there was a remarkable difference in expression between tumor and normal samples (Figure 3C). In addition, the expression of LINC01572 and TMPO-AS1 differed significantly between tumor stages, with higher expression associated with higher tumor stage (*p* < .05) (Figure 3D). Survival curves illustrated the prognostic value of each signature FMRlncRNAs (Figure 3E).

### Single-cell RNA analysis

**FIGURE 4:**
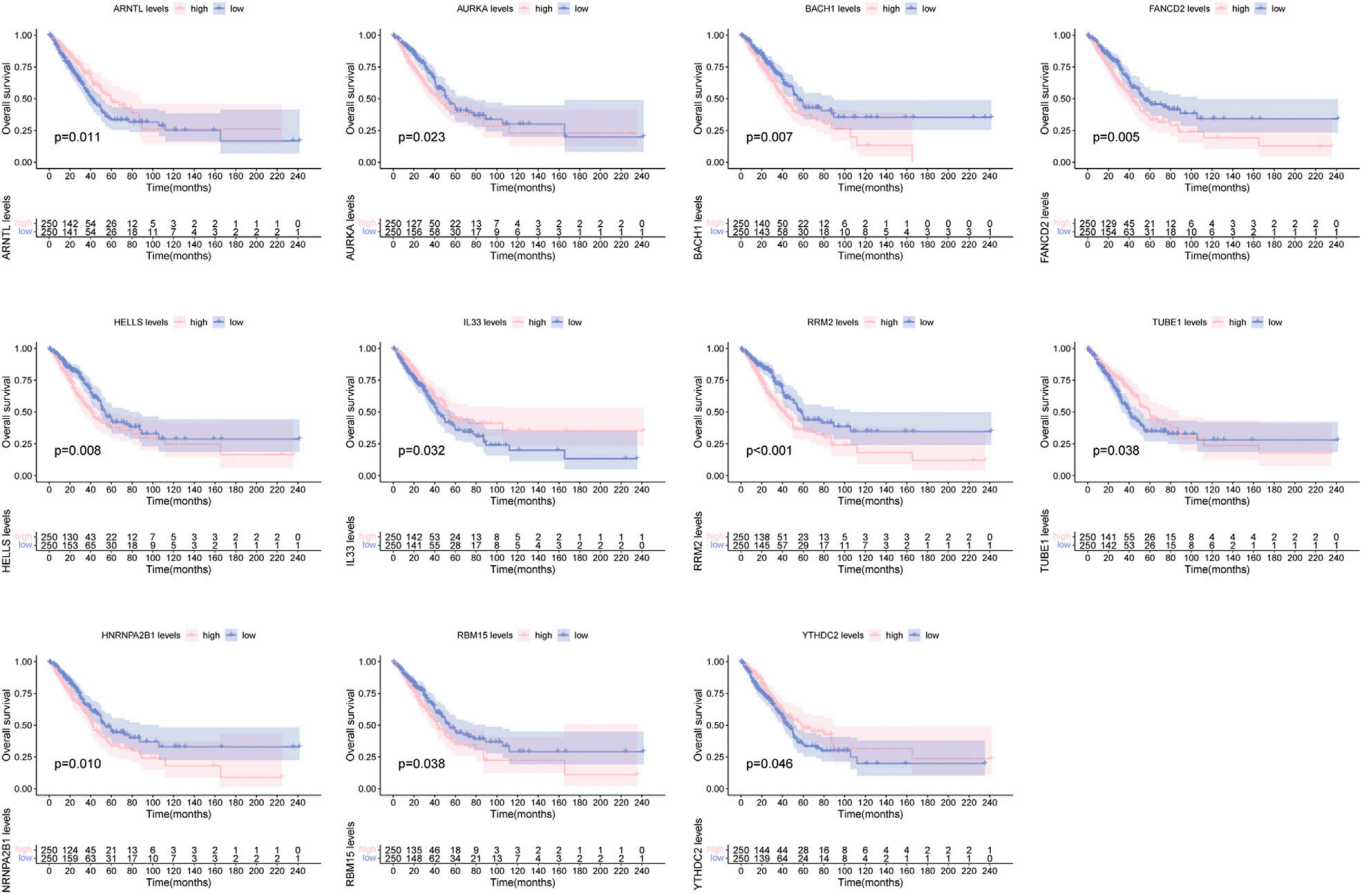
Survival curve of clinical survival for patients between groups based on 11 ferroptosis and m6A related genes.

There were 26 FRGs and 6 MRGs associated with the 5 lncRNAs involved in the signature (Supplementary Table S2). Survival curves revealed the prognostic value of 11 genes. Among them, patients with high expression of ARNTL, IL33, TUBE1, YTHDC2 displayed a favorable outcome, while cases with high expression of AURKA, BACH1, FANCD2, HELLS, RRM2, HNRNPA2B1, RBM15 had a dismal clinical outcome (Figure 4).

**FIGURE 5:**
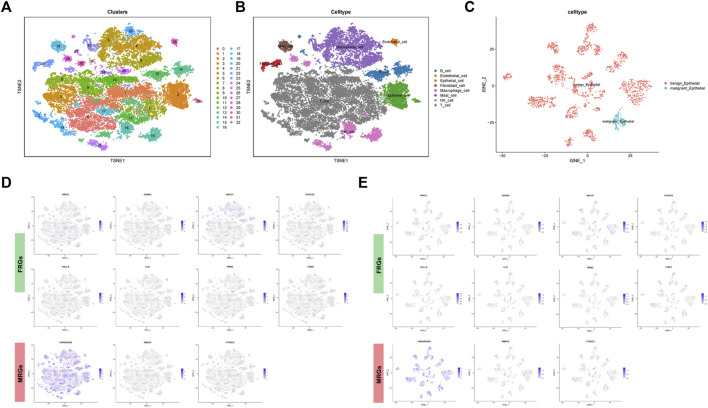
Single cell sequencing analysis. **(A)** Dimensionality reduction and cluster analysis. **(B)** Cell population annotation. **(C)** Classification of epithelial cells into benign and malignant epithelial cells. **(D)** Cellular location of FRGs and MRGs in all cells and in **(E)** epithelial cells.

In order to unearth the cellular location of above 11 genes, single-cell RNA analysis was applied. The GSE123904 dataset was first divided into 33 cell clusters (Figure 5A). In Figure 5B, a total of eight types of cell subpopulation were determined based on cell markers. In addition, we analyzed the malignancy of the epithelial cells (Figure 5C). The cellular location landscape of 11 genes in all cell population and epithelial cells was shown as in Figures 5D, E.

### Prognostic performance of FMRLM

**FIGURE 6:**
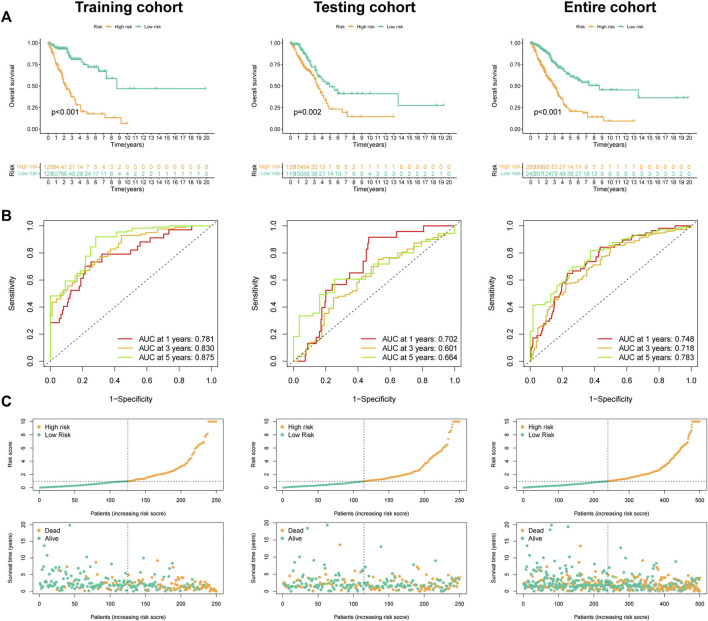
Predictive ability of the FMRLM. **(A)** Survival analysis between two risk groups in the train, test, and all sets, respectively. **(B)** ROC curves analysis. **(C)** Exhibition of FMRLM on risk score and survival status between two groups in three cohorts.

In Figure 6A, the high-FMRLM group presented a dismal outcome in three LUAD cohorts (Figure 6A). In terms of AUC, the 1-, 3-, and 5-year AUCs were .781, .830, and .875 for the train set, .702, .601, and .664 for the test set, and .748, .718 and .783 for the whole group, respectively (Figure 6B), and our risk scores distinguish patients well (Figure 6C).

### Independent prognosis analysis of FMRLM

**FIGURE 7:**
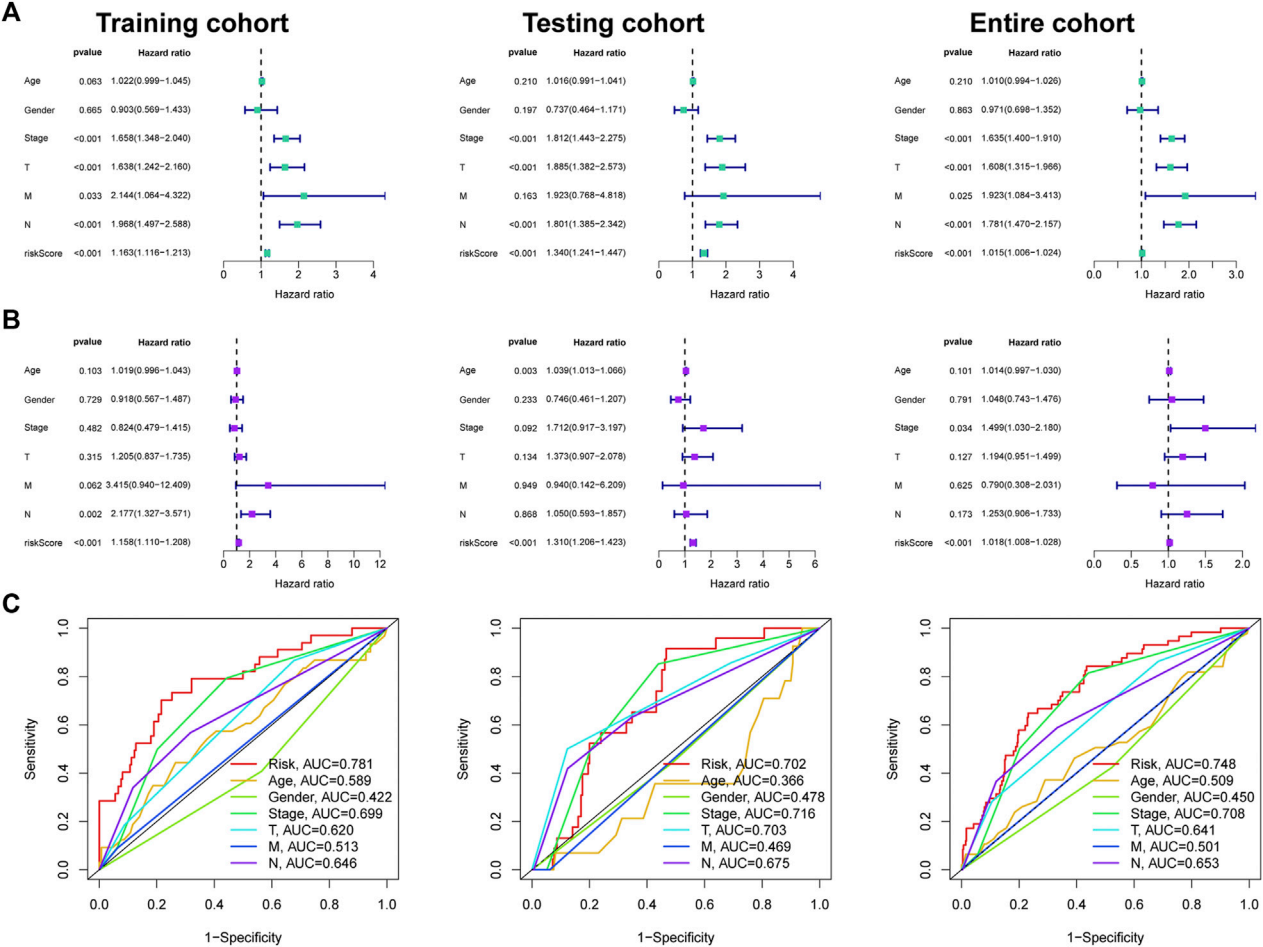
Independent prognosis analysis. **(A)** Univariate and **(B)** multivariate Cox regression analysis of clinical factors and risk score with survival outcome. **(C)** ROC curves analysis.

Univariate Cox regression disclosed that FMRLM was greatly meaningful in three cohorts (Figure 7A). After employing multivariate regression, the FMRLM was also an independent prognostic index of LUAD (Figure 7B). ROC curves illustrated the FMRLM had better predictive ability than other clinical variables (Figure 7C).

### Immune microenvironment analysis

**FIGURE 8:**
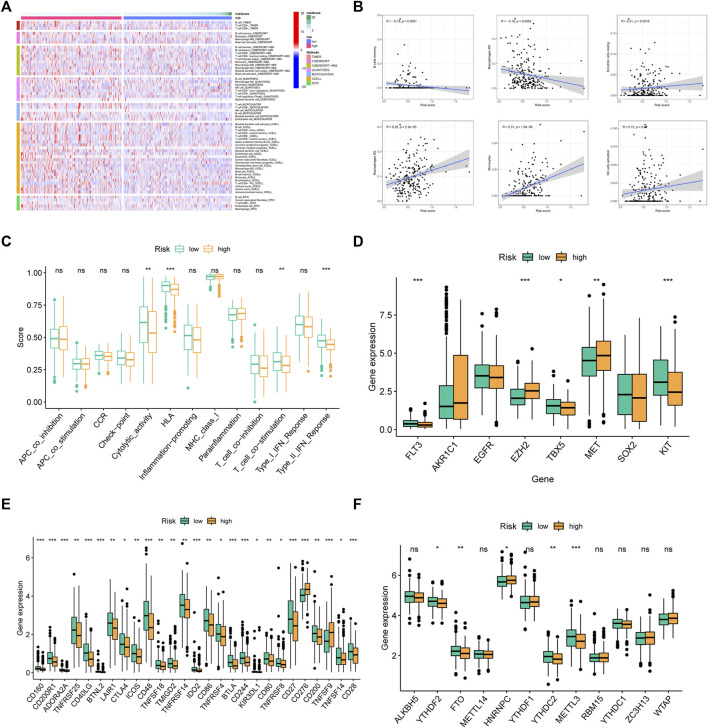
Immune microenvironment analysis. **(A)** The immune cell differential expression analysis of risk groups. **(B)** The correlation between risk score and immune cells. **(C)** Analysis of differences in immune functions, **(D)** chemosensitivity-related genes, **(E)** immune checkpoints, and **(F)** m6A regulators between risk groups (ns > .05, *p < .05, **p < .01, ***p < .001).

We analyzed the infiltration level of immunocytes of two groups using data from several platforms (Figure 8A). The results showed that B cells memory and Macrophages M0 were enriched in low-FMRLM group, while dendritic cells resting, Macrophages M2, Monocytes, and NK cells activated were enriched in high-FMRLM group (Figure 8B). Also, we observed that the low-FMRLM group had more prosperous immune functions such as cytolytic activity, HLA, T cell co-stimulation, and type II IFN response (Figure 8C), and several genes associated with sensitivity to radiotherapies such as FLT3, EZH2, TBX5, MET, and KIT had different expression between two subgroups (Figure 8D). CD160 and CTLA4 are highly expressed in the low-FMRLM group, while CD276 and TNFSF9 are more highly expressed in the high-FMRLM group (Figure 8E), and the expression of several m6A regulators (YTHDF2, FTO, HNRNPC, YTHDC2, and METTL3) differed between two subgroups (Figure 8F).

### Drug sensitivity analysis

**FIGURE 9:**
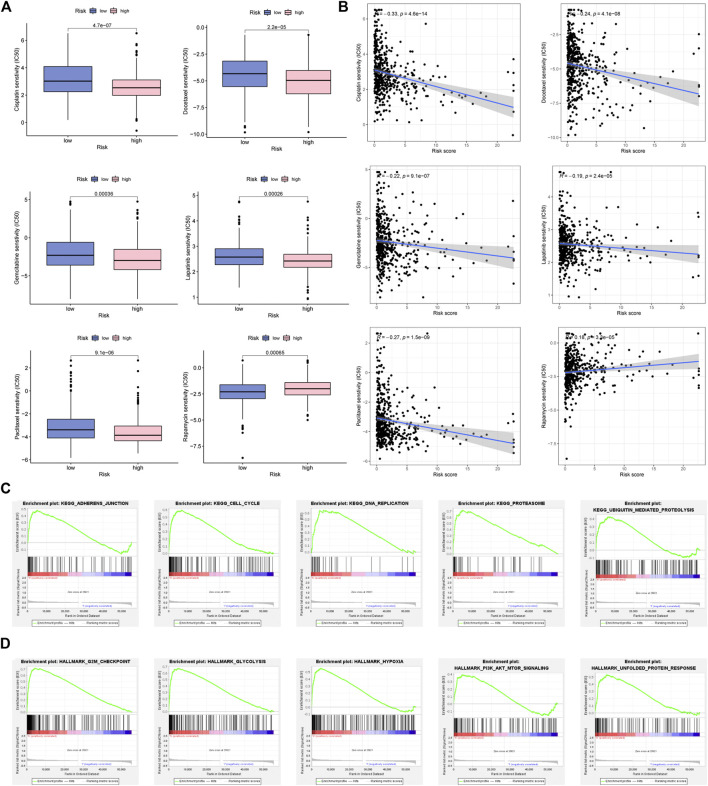
Drug sensitivity analysis and GSEA enrichment. **(A)** Differences and **(B)** correlation analysis between risk score and drug IC50. **(C)** KEGG and **(D)** Hallmark enrichment.

As revealed in Figure 9, Cisplatin, Docetaxel, Gemcitabine, Lapatinib, and Paclitaxel had a higher IC50 in low-FMRLM group, and their IC50 values were negatively correlated with the risk score, suggesting that patients with high risk were more sensitive to them. Rapamycin, on the other hand, had a higher IC50 in high-FMRLM group (Figures 9A, B).

### GSEA of FMRLM

To unearth the underlying function and pathways of signature, GSEA enrichment was conducted. In Figure 9C, we observed that cell cycle, DNA repair and ubiquitination were greatly enriched in high-risk cohort. In terms of Hallmark in tumor, glycolysis, hypoxia and PI3K/AKT/MTOR were activated in high-risk cohort (Figure 9D).

### Downregulation of LINC01572 induced cell ferroptosis

**FIGURE 10:**
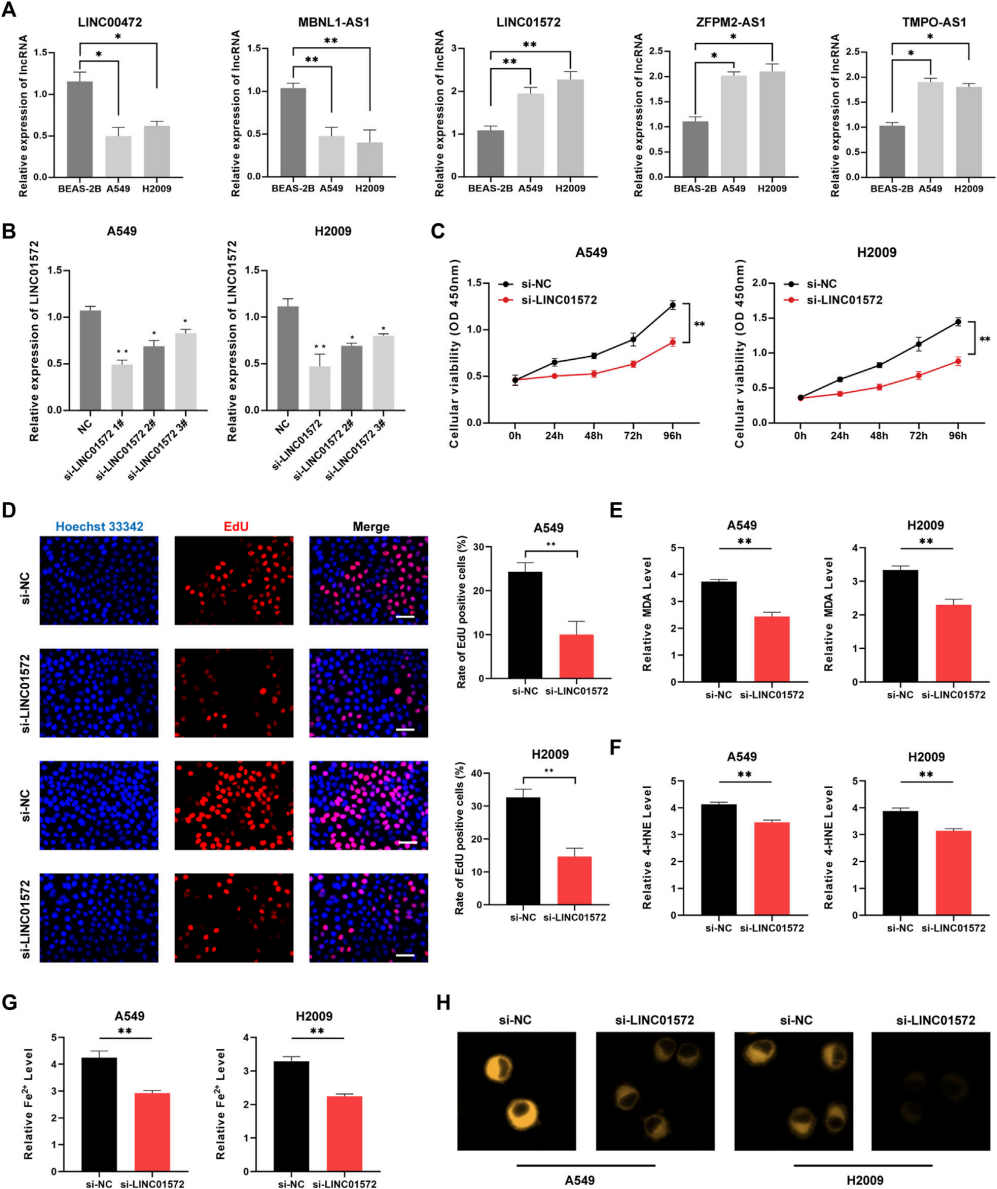
Downregulation of LINC01572 induced cell ferroptosis. **(A)** Expression patterns of five lncRNAs in different cell lines by qRT-PCR. **(B)** LINC01572 A was successfully knocked down in LUAD cell lines **(C)** CCK8 assay, **(D)** EdU assay, **(E)** MDA assay, **(F)** 4HNE assay and **(G,H)** cellular iron assay in different treatment groups (*p < .05, ***p < .001).

PCR results suggested that LINC00472 and MBNL1-AS1 presented lower expression in LUAD cell lines, whereas LINC01572, ZFPM2-AS1, and TMPO-AS1 were highly expressed in A549 and H2009 cell lines (Figure 10A). Figure 10B illustrated favorable knock-down efficiency of LINC01572 in two cell lines. CCK8 assay indicated a remarkable decline in the cell viability with si-LINC01572 compared to the NC group (Figure 10C), with the same similar results for EdU assay (Figure 10D). Based on the cellular MDA, 4HNE and iron assays, we observed that silencing LINC01572 could promote cell ferroptosis of A549 and H2009 cell lines (Figures 10E–H).

## Discussion

Iron ions play a central part in the facilitation of the process of ferroptosis as the most essential nutrient for tumor cell survival. Consequently, anti-tumor by inducing cellular ferroptosis has become a hot research topic in recent years. RNA methylation has recently been reported to regulate ferroptosis in gastric cancer, suggesting combination of methylation modification and ferroptosis might be therapy target in tumor management (Yang et al., 2022).

The risk signature established in this study consists of 5 lncRNAs, among which LINC01572, TMPO-AS1 and ZFPM2-AS1 are risk lncRNAs, and LINC00472 and MBNL1-AS1 are protective lncRNAs. There is a lack of systematic studies of LINC01572 in LUAD. As suggested by Chen et al. (2017), LINC01572 is upregulation in LC and its expression level can distinguish between early and advanced stage. The expression of LINC01572 in the blood of cisplatin resistant gastric cancer patients is significantly increased, and it may produce chemotherapy resistance through the mechanism of inducing autophagy (Song et al., 2020). Numerous reports demonstrated that TMPO-AS1 has been revealed to be a key biomarker for evaluating the prognosis of LUAD (Li et al., 2016; Wang et al., 2019). ZFPM2-AS1 is an oncogene in gastric cancer (Sasa et al., 2022), but it has also been shown to be involved in the regulation of LUAD cell growth, which can be used as a new potential target for LUAD treatment (Han et al., 2020). As a potential lncRNA in human LUAD, LINC00472 has been proved to be a tumor suppressor, which could suppress LUAD cells viability (Sui et al., 2016). MBNL1-AS1 is a crucial tumor regulator and plays a negative regulatory role in a variety of tumors including lung cancer (Cao et al., 2020).

Immunosuppressive cells such as tumor-associated macrophages (TAM), cancer-associated fibroblasts (CAF), and neutrophils can be tumor-modified to produce a tumor-supportive microenvironment (Quail and Joyce, 2013; Chu et al., 2021). In our research, patients with high risk had significantly higher proportions of immunosuppressive cells. Macrophages act as scavengers, regulating the immune response to pathogens and maintaining tissue homeostasis. Immunotherapies and therapeutic strategies aimed at reducing the proportion of M2 macrophages or converting M2 macrophages to M1 macrophages have been proposed to suppress tumor survival (Sica et al., 2006). Several studies have shown that CAFs can promote tumor growth in several ways: secreting ECM proteins, inducing inflammation and angiogenesis, altering the metabolism and epigenome of cancer cells, establishing immunosuppression, conferring therapeutic resistance, and radiation protection (Mhaidly and Mechta-Grigoriou, 2021). Neutrophils are important intrinsic immune cells for the body’s antibacterial defense. In recent years, an elevated neutrophil-to-lymphocyte ratio has been recognized as a poor prognostic indicator of overall survival in cancer patients. Neutrophils form a sticky reticulum called neutrophil extracellular trap (NET) that has been shown to be involved in tumor metastasis (Erpenbeck and Schön, 2017).

One extremely promising approach to achieving tumor immunotherapy is to block the immune checkpoint by which tumor disguise themselves as normal cells. To date, immune checkpoint blocking drugs targeting CTLA-4 and PD-L1 have been used in the clinic and represent a milestone in antitumor therapy (Topalian et al., 2016). In the present study, CD27 was significantly less expressed in the low-FMRLM group. CD27 is a member of the tumor necrosis factor receptor superfamily and, in combination with its natural ligand CD70, activates the differentiation of T cells into effector and memory T cells and thus has potential as an immunomodulatory target in cancer therapy (Starzer and Berghoff, 2020). Moreover, among the immune checkpoints we examined, CD276 and CD28 belong to the B7 and CD28 families, representing immune signaling of tumors and immune cells, respectively.

The human leukocyte antigen (HLA) is a highly genetically polymorphic group of closely linked genes that control intercellular recognition and regulate the immune response. As an independent factor in tumor-associated antigen presentation, HLA-I plays an important role in antitumor immune response and neoplastic tumor progression. CD8+T cell-dependent killing of cancer cells requires HLA-I molecules for efficient tumor antigen presentation (Chowell et al., 2018). The absence of HLA class I molecules on the tumor cell surface is a major obstacle to the success of T cell-mediated immunotherapy (Garrido, 2019). The interferon-stimulated response element (ISRE) of all classical HLA-I genes mediates IFN-γ-induced transactivation, and of the non-classical HLA-I molecules, only the ISRE of HLA-F mediates IFN-γ induction (Gobin et al., 1999).

As is known, m6A modification is one of the emerging frontiers of research, and its modifying function has been linked to the development and progression of many human diseases, including lung cancer (Wang et al., 2020). In our m6A regulator expression analysis, METTL3, FTO, and YTHDC2 were significantly differentially expressed among two groups. It has also been shown that these three m6A regulators are associated with LUAD growth and prognosis. METTL3, YTHDC2 are upregulated in LUAD and promote LUAD growth (Zhang et al., 2020; Zhang et al., 2022a; Xu et al., 2022). Downregulation of YTHDC2 is associated with poor clinical outcome (Ma et al., 2021). As revealed by Ning et al. (2022), FTO is lowly expressed in poor prognosis LUAD samples and has predominantly antitumor activity.

To further test the speculation, we analyzed the pattern of Chemoradiotherapy sensitivity genes. Our data suggested FLT3 and KIT were upregulated in low-FMRLM group. It has been shown that Imatinib mesylate treatment of advanced melanoma yielded significant clinical responses in patients with KIT gene mutations (Carvajal et al., 2011). SNHG17 epigenetically represses LATS2 expression by recruiting EZH2 to the promoter region of LATS2, exacerbating the malignant phenotype of gefitinib-resistant LUAD cells (Zhang et al., 2022b). CAPN1 could inhibit the stability of c-Met, which in turn confer chemotherapy resistance to LUAD cells (Chen et al., 2020).

In GSEA enrichment studies between two groups were enriched for the characteristics of malignancy: Glycolysis, Hypoxia, PI3K/AKT/MTOR signaling. PI3K signaling pathway is essential for cell growth Overactivated in many cancer types, possible mechanisms by which the PI3K/AKT/mTOR axis promotes oncogenic transformation include stimulation of proliferation, survival, metabolic reprogramming, invasion, metastasis, inhibition of autophagy and senescence (Liu et al., 2022d). Glycolysis is increasingly being revealed as a marker of tumor progression. The possible pro-cancer mechanism is that induced glycolysis and increased glucose uptake promote lipid, protein, and nucleotide production, thereby promoting tumor cell proliferation and division. Multiple genes associated with glycolysis have been reported to be involved in cancer progression and LUAD is no exception (Zhou et al., 2019). Similarly, hypoxia has been identified as a factor in tumor progression and poor prognosis. Interestingly, hypoxia induces a metabolic shift from oxidative phosphorylation to glycolysis and increases glycogen synthesis, and this metabolic reprogramming favors tumor growth (Li et al., 2020).

Nevertheless, there are still several issues to be addressed. First, the signature was created according to patient information downloaded from public databases, which has the disadvantage of being limited and incomplete, and the restriction of selection bias. Second, our study lacks further validation with wet lab experiments.

## Conclusion

In summary, we successfully created a robust risk score system based on FMRncRNAs. Our data highlights the prognostic value and possible clinical potency of FMRLM, which may serve as the therapeutic target for LUAD.

## Data Availability

The original contributions presented in the study are included in the article/Supplementary Material, further inquiries can be directed to the corresponding authors.

## References

[B1] Bray F. Ferlay J. Soerjomataram I. Siegel R. L. Torre L. A. Jemal A. (2018). Global cancer statistics 2018: GLOBOCAN estimates of incidence and mortality worldwide for 36 cancers in 185 countries. CA Cancer J. Clin. 68, 394–424. 10.3322/caac.21492 30207593

[B2] Cao G. Tan B. Wei S. Shen W. Wang X. Chu Y. (2020). Down-regulation of MBNL1-AS1 contributes to tumorigenesis of NSCLC via sponging miR-135a-5p. Biomed. Pharmacother. 125, 109856. 10.1016/j.biopha.2020.109856 32092823

[B3] Carvajal R. D. Antonescu C. R. Wolchok J. D. Chapman P. B. Roman R-A. Teitcher J. (2011). KIT as a therapeutic target in metastatic melanoma. JAMA 305, 2327–2334. 10.1001/jama.2011.746 21642685 PMC3986039

[B4] Chen F. Song J. Ye Z. Xu B. Cheng H. Zhang S. (2021). Integrated analysis of cell cycle-related and immunity-related biomarker signatures to improve the prognosis prediction of lung adenocarcinoma. Front. Oncol. 11, 666826. 10.3389/fonc.2021.666826 34150632 PMC8212041

[B5] Chen W-J. Tang R-X. He R-Q. Li D-Y. Liang L. Zeng J-H. (2017). Clinical roles of the aberrantly expressed lncRNAs in lung squamous cell carcinoma: A study based on RNA-sequencing and microarray data mining. Oncotarget 8, 61282–61304. 10.18632/oncotarget.18058 28977863 PMC5617423

[B6] Chen Y. Tang J. Lu T. Liu F. (2020). CAPN1 promotes malignant behavior and erlotinib resistance mediated by phosphorylation of c-Met and PIK3R2 via degrading PTPN1 in lung adenocarcinoma. Thorac. Cancer 11, 1848–1860. 10.1111/1759-7714.13465 32395869 PMC7327690

[B7] Chowell D. Morris L. G. T. Grigg C. M. Weber J. K. Samstein R. M. Makarov V. (2018). Patient HLA class I genotype influences cancer response to checkpoint blockade immunotherapy. Science 359, 582–587. 10.1126/science.aao4572 29217585 PMC6057471

[B8] Chu Z-Q. Zhang K-C. Chen L. (2021). Neutrophil extracellular traps in gastrointestinal cancer. World J. Gastroenterol. 27, 5474–5487. 10.3748/wjg.v27.i33.5474 34588746 PMC8433615

[B9] Coudray N. Ocampo P. S. Sakellaropoulos T. Narula N. Snuderl M. Fenyö D. (2018). Classification and mutation prediction from non-small cell lung cancer histopathology images using deep learning. Nat. Med. 24, 1559–1567. 10.1038/s41591-018-0177-5 30224757 PMC9847512

[B10] Cui Y. Zhang C. Ma S. Li Z. Wang W. Li Y. (2021). RNA m6A demethylase FTO-mediated epigenetic up-regulation of LINC00022 promotes tumorigenesis in esophageal squamous cell carcinoma. J. Exp. Clin. Cancer Res. 40, 294. 10.1186/s13046-021-02096-1 34544449 PMC8451109

[B11] Denisenko T. V. Budkevich I. N. Zhivotovsky B. (2018). Cell death-based treatment of lung adenocarcinoma. Cell Death Dis. 9, 117. 10.1038/s41419-017-0063-y 29371589 PMC5833343

[B12] Du J. Ji H. Ma S. Jin J. Mi S. Hou K. (2021). m6A regulator-mediated methylation modification patterns and characteristics of immunity and stemness in low-grade glioma. Brief. Bioinform 22, bbab013. 10.1093/bib/bbab013 33594424

[B13] Erpenbeck L. Schön M. P. (2017). Neutrophil extracellular traps: Protagonists of cancer progression? Oncogene 36, 2483–2490. 10.1038/onc.2016.406 27941879

[B14] Garrido F. (2019). HLA class-I expression and cancer immunotherapy. Adv. Exp. Med. Biol. 1151, 79–90. 10.1007/978-3-030-17864-2_3 31140107

[B15] Geng R. Song J. Zhong Z. Ni S. Liu W. He Z. (2022). Crosstalk of redox-related subtypes, establishment of a prognostic model and immune responses in endometrial carcinoma. Cancers (Basel) 14, 3383. 10.3390/cancers14143383 35884444 PMC9319597

[B16] Gobin S. J. van Zutphen M. Woltman A. M. van den Elsen P. J. (1999). Transactivation of classical and nonclassical HLA class I genes through the IFN-stimulated response element. J. Immunol. 163, 1428–1434. 10415043

[B17] Gu H. Song J. Chen Y. Wang Y. Tan X. Zhao H. (2022). Inflammation-related LncRNAs signature for prognosis and immune response evaluation in uterine corpus endometrial carcinoma. Front. Oncol. 12, 923641. 10.3389/fonc.2022.923641 35719911 PMC9201290

[B18] Gupta R. A. Shah N. Wang K. C. Kim J. Horlings H. M. Wong D. J. (2010). Long non-coding RNA HOTAIR reprograms chromatin state to promote cancer metastasis. Nature 464, 1071–1076. 10.1038/nature08975 20393566 PMC3049919

[B19] Han S. Cao D. Sha J. Zhu X. Chen D. (2020). LncRNA ZFPM2-AS1 promotes lung adenocarcinoma progression by interacting with UPF1 to destabilize ZFPM2. Mol. Oncol. 14, 1074–1088. 10.1002/1878-0261.12631 31919993 PMC7191191

[B20] He L. Li H. Wu A. Peng Y. Shu G. Yin G. (2019). Functions of N6-methyladenosine and its role in cancer. Mol. Cancer 18, 176. 10.1186/s12943-019-1109-9 31801551 PMC6892141

[B21] Imielinski M. Berger A. H. Hammerman P. S. Hernandez B. Pugh T. J. Hodis E. (2012). Mapping the hallmarks of lung adenocarcinoma with massively parallel sequencing. Cell 150, 1107–1120. 10.1016/j.cell.2012.08.029 22980975 PMC3557932

[B22] Li D-S. Ainiwaer J-L. Sheyhiding I. Zhang Z. Zhang L-W. (2016). Identification of key long non-coding RNAs as competing endogenous RNAs for miRNA-mRNA in lung adenocarcinoma. Eur. Rev. Med. Pharmacol. Sci. 20, 2285–2295. 27338053

[B23] Li L. Yang L. Fan Z. Xue W. Shen Z. Yuan Y. (2020). Hypoxia-induced GBE1 expression promotes tumor progression through metabolic reprogramming in lung adenocarcinoma. Signal Transduct. Target Ther. 5, 54. 10.1038/s41392-020-0152-8 32439898 PMC7242448

[B24] Liu C. Wang Y. Dao Y. Wang S. Hou F. Yang Z. (2022). Upregulation of CENPM facilitates lung adenocarcinoma progression via PI3K/AKT/mTOR signaling pathway. Acta Biochim. Biophys. Sin. (Shanghai) 54, 99–112. 10.3724/abbs.2021013 35130633 PMC9909302

[B25] Liu J. Geng R. Ni S. Cai L. Yang S. Shao F. (2022). Pyroptosis-related lncRNAs are potential biomarkers for predicting prognoses and immune responses in patients with UCEC. Mol. Ther. Nucleic Acids 27, 1036–1055. 10.1016/j.omtn.2022.01.018 35228898 PMC8844853

[B26] Liu J. Geng R. Zhong Z. Zhang Y. Ni S. Liu W. (2022). N1-Methyladenosine-Related lncRNAs are potential biomarkers for predicting prognosis and immune response in uterine corpus endometrial carcinoma. Oxid. Med. Cell Longev. 2022, 2754836. 10.1155/2022/2754836 35965688 PMC9372539

[B27] Liu J. Mei J. Wang Y. Chen X. Pan J. Tong L. (2021). Development of a novel immune-related lncRNA signature as a prognostic classifier for endometrial carcinoma. Int. J. Biol. Sci. 17, 448–459. 10.7150/ijbs.51207 33613104 PMC7893582

[B28] Liu Y. Shi M. He X. Cao Y. Liu P. Li F. (2022). LncRNA-PACERR induces pro-tumour macrophages via interacting with miR-671-3p and m6A-reader IGF2BP2 in pancreatic ductal adenocarcinoma. J. Hematol. Oncol. 15, 52. 10.1186/s13045-022-01272-w 35526050 PMC9077921

[B29] Ma L. Zhang X. Yu K. Xu X. Chen T. Shi Y. (2021). Targeting SLC3A2 subunit of system XC- is essential for m6A reader YTHDC2 to be an endogenous ferroptosis inducer in lung adenocarcinoma. Free Radic. Biol. Med. 168, 25–43. 10.1016/j.freeradbiomed.2021.03.023 33785413

[B30] Ma S. Chen C. Ji X. Liu J. Zhou Q. Wang G. (2019). The interplay between m6A RNA methylation and noncoding RNA in cancer. J. Hematol. Oncol. 12, 121. 10.1186/s13045-019-0805-7 31757221 PMC6874823

[B31] Ma Y. Zhang J. Wen L. Lin A. (2018). Membrane-lipid associated lncRNA: A new regulator in cancer signaling. Cancer Lett. 419, 27–29. 10.1016/j.canlet.2018.01.008 29330108

[B32] Mangiola S. Doyle M. A. Papenfuss A. T. (2021). Interfacing Seurat with the R tidy universe. Bioinformatics 37, 4100–4107. 10.1093/bioinformatics/btab404 34028547 PMC9502154

[B33] Mhaidly R. Mechta-Grigoriou F. (2021). Role of cancer-associated fibroblast subpopulations in immune infiltration, as a new means of treatment in cancer. Immunol. Rev. 302, 259–272. 10.1111/imr.12978 34013544 PMC8360036

[B34] Mou Y. Wang J. Wu J. He D. Zhang C. Duan C. (2019). Ferroptosis, a new form of cell death: Opportunities and challenges in cancer. J. Hematol. Oncol. 12, 34. 10.1186/s13045-019-0720-y 30925886 PMC6441206

[B35] Ning J. Wang F. Bu J. Zhu K. Liu W. (2022). Down-regulated m6A reader FTO destabilizes PHF1 that triggers enhanced stemness capacity and tumor progression in lung adenocarcinoma. Cell Death Discov. 8, 354. 10.1038/s41420-022-01125-y 35945194 PMC9363432

[B36] Peng W-X. Koirala P. Mo Y-Y. (2017). LncRNA-mediated regulation of cell signaling in cancer. Oncogene 36, 5661–5667. 10.1038/onc.2017.184 28604750 PMC6450570

[B37] Quail D. F. Joyce J. A. (2013). Microenvironmental regulation of tumor progression and metastasis. Nat. Med. 19, 1423–1437. 10.1038/nm.3394 24202395 PMC3954707

[B38] Ritchie M. E. Phipson B. Wu D. Hu Y. Law C. W. Shi W. (2015). Limma powers differential expression analyses for RNA-sequencing and microarray studies. Nucleic Acids Res. 43, e47. 10.1093/nar/gkv007 25605792 PMC4402510

[B39] Sasa G. B. K. Xuan C. Lyu G. Ding X. Meiyu F. (2022). Long non-coding RNA ZFPM2-AS1: A novel biomarker in the pathogenesis of human cancers. Mol. Biotechnol. 64, 725–742. 10.1007/s12033-021-00443-3 35098483

[B40] Shen M. Li Y. Wang Y. Shao J. Zhang F. Yin G. (2021). N6-methyladenosine modification regulates ferroptosis through autophagy signaling pathway in hepatic stellate cells. Redox Biol. 47, 102151. 10.1016/j.redox.2021.102151 34607160 PMC8495178

[B41] Sica A. Schioppa T. Mantovani A. Allavena P. (2006). Tumour-associated macrophages are a distinct M2 polarised population promoting tumour progression: Potential targets of anti-cancer therapy. Eur. J. Cancer 42, 717–727. 10.1016/j.ejca.2006.01.003 16520032

[B42] Song J. Liu Y. Guan X. Zhang X. Yu W. Li Q. (2021). A novel ferroptosis-related biomarker signature to predict overall survival of esophageal squamous cell carcinoma. Front. Mol. Biosci. 8, 675193. 10.3389/fmolb.2021.675193 34291083 PMC8287967

[B43] Song J. Sun Y. Cao H. Liu Z. Xi L. Dong C. (2021). A novel pyroptosis-related lncRNA signature for prognostic prediction in patients with lung adenocarcinoma. Bioengineered 12, 5932–5949. 10.1080/21655979.2021.1972078 34488540 PMC8806662

[B44] Song Z. Jia N. Li W. Zhang X-Y. (2020). LINC01572 regulates cisplatin resistance in gastric cancer cells by mediating miR-497-5p. Onco Targets Ther. 13, 10877–10887. 10.2147/OTT.S267915 33149605 PMC7602899

[B45] Starzer A. M. Berghoff A. S. (2020). New emerging targets in cancer immunotherapy: CD27 (TNFRSF7). ESMO Open 4, e000629. 10.1136/esmoopen-2019-000629 32152062 PMC7082637

[B46] Sui J. Li Y-H. Zhang Y-Q. Li C-Y. Shen X. Yao W-Z. (2016). Integrated analysis of long non-coding RNA-associated ceRNA network reveals potential lncRNA biomarkers in human lung adenocarcinoma. Int. J. Oncol. 49, 2023–2036. 10.3892/ijo.2016.3716 27826625

[B47] Topalian S. L. Taube J. M. Anders R. A. Pardoll D. M. (2016). Mechanism-driven biomarkers to guide immune checkpoint blockade in cancer therapy. Nat. Rev. Cancer 16, 275–287. 10.1038/nrc.2016.36 27079802 PMC5381938

[B48] Wang T. Kong S. Tao M. Ju S. (2020). The potential role of RNA N6-methyladenosine in Cancer progression. Mol. Cancer 19, 88. 10.1186/s12943-020-01204-7 32398132 PMC7216508

[B49] Wang Y. Fu J. Wang Z. Lv Z. Fan Z. Lei T. (2019). Screening key lncRNAs for human lung adenocarcinoma based on machine learning and weighted gene co-expression network analysis. Cancer Biomark. 25, 313–324. 10.3233/CBM-190225 31322548 PMC12828841

[B50] Wang Z. Wang X. Zhang T. Su L. Liu B. Zhu Z. (2021). LncRNA MALAT1 promotes gastric cancer progression via inhibiting autophagic flux and inducing fibroblast activation. Cell Death Dis. 12, 368. 10.1038/s41419-021-03645-4 33824303 PMC8024309

[B51] Wilkerson M. D. Hayes D. N. (2010). ConsensusClusterPlus: A class discovery tool with confidence assessments and item tracking. Bioinformatics 26, 1572–1573. 10.1093/bioinformatics/btq170 20427518 PMC2881355

[B52] Xu Y. Lv D. Yan C. Su H. Zhang X. Shi Y. (2022). METTL3 promotes lung adenocarcinoma tumor growth and inhibits ferroptosis by stabilizing SLC7A11 m6A modification. Cancer Cell Int. 22, 11. 10.1186/s12935-021-02433-6 34996469 PMC8742440

[B53] Yang H. Hu Y. Weng M. Liu X. Wan P. Hu Y. (2022). Hypoxia inducible lncRNA-CBSLR modulates ferroptosis through m6A-YTHDF2-dependent modulation of CBS in gastric cancer. J. Adv. Res. 37, 91–106. 10.1016/j.jare.2021.10.001 35499052 PMC9039740

[B54] Zhang D. Zhang D. Wang C. Yang X. Zhang R. Li Q. (2022). Gene and prognostic value of N6-methyladenosine (m6A) modification regulatory factors in lung adenocarcinoma. Eur. J. Cancer Prev. 31, 354–362. 10.1097/CEJ.0000000000000717 34519693

[B55] Zhang H. Wang S-Q. Wang L. Lin H. Zhu J-B. Chen R. (2022). m6A methyltransferase METTL3-induced lncRNA SNHG17 promotes lung adenocarcinoma gefitinib resistance by epigenetically repressing LATS2 expression. Cell Death Dis. 13, 657. 10.1038/s41419-022-05050-x 35902569 PMC9334586

[B56] Zhang Y. Liu X. Liu L. Li J. Hu Q. Sun R. (2020). Expression and prognostic significance of m6A-related genes in lung adenocarcinoma. Med. Sci. Monit. 26, e919644. 10.12659/MSM.919644 32086933 PMC7049251

[B57] Zhou J. Zhang S. Chen Z. He Z. Xu Y. Li Z. (2019). CircRNA-ENO1 promoted glycolysis and tumor progression in lung adenocarcinoma through upregulating its host gene ENO1. Cell Death Dis. 10, 885. 10.1038/s41419-019-2127-7 31767835 PMC6877563

