# Supplementary material for: Construction of a ferroptosis scoring system and identification of LINC01572 as a novel ferroptosis suppressor in lung adenocarcinoma
Source: Front Pharmacol. 2023 Jan 4;13:1098136. doi: 10.3389/fphar.2022.1098136 (PMC9846555; doi:10.3389/fphar.2022.1098136)
Supplement: Supplementary file 1 [file Table1.DOCX]

Supplementary Table 1 Primer and siRNA sequences for each gene.

| Gene | Primer sequence | |
| --- | --- | --- |
| GAPDH | Forward | 5′-CTGGGCTACACTGAGCACC-3′ |
|  | Reverse | 5′-AAGTGGTCGTTGAGGGCAATG-3′ |
| LINC00472 | Forward | 5′-GATGGCAGCTGTCTCTCTCC-3′ |
|  | Reverse | 5′-GGGCCTCTCTGACCGTATCT-3' |
| LINC01572 | Forward | 5’-GGAAGGACACCATTGACAGC-3’ |
|  | Reverse | 5’-GGTTGGTGCCTGACTTTTGT-3’ |
| MBNL-AS1 | Forward | 5’- GGGTGAATGAGAGTGTGGGA-3’ |
|  | Reverse | 5’- ATCACAGACAATGGGAGGCA-3’ |
| ZFPM2-AS1 | Forward | 5’-GGTGGCACCTGAAATCACAGA-3’ |
|  | Reverse | 5’-TGCAAGATGACGCTCAGTCG-3’ |
| TMPO-AS1 | Forward | 5’- AGCCCACACACTACAGGCAG-3’ |
|  | Reverse | 5’-GCACAAAAGCAGTACGACCTA-3’ |
| Gene | siRNA-LINC01572 sequence | |
| siRNA1 | 5′-GCCUGAAGUACACGUGGAAAU-3′ | |
| siRNA2 | 5′-GCUUGGUCAUUGAGAUCUACU-3′ | |
| siRNA3 | 5′-GGCAAACCUGGAACAGUAACC-3′ | |
