# Supplementary material for: Construction of a ferroptosis scoring system and identification of LINC01572 as a novel ferroptosis suppressor in lung adenocarcinoma
Source: Front Pharmacol. 2023 Jan 4;13:1098136. doi: 10.3389/fphar.2022.1098136 (PMC9846555; doi:10.3389/fphar.2022.1098136)
Supplement: Supplementary file 2 [file Table2.DOCX]

Supplementary Table 2 26 FRGs and 6 MRGs associated with the 5 lncRNAs.

26 FRGs

| HELLS | LINC01572 |
| --- | --- |
| ANGPTL7 | LINC01572 |
| ZFP69B | LINC01572 |
| TUBE1 | LINC01572 |
| IREB2 | LINC01572 |
| GABPB1 | LINC01572 |
| PIK3CA | LINC01572 |
| MAPK8 | LINC01572 |
| ATM | LINC01572 |
| FANCD2 | TMPO-AS1 |
| HELLS | TMPO-AS1 |
| TFRC | TMPO-AS1 |
| STMN1 | TMPO-AS1 |
| RRM2 | TMPO-AS1 |
| AURKA | TMPO-AS1 |
| ANGPTL7 | ZFPM2-AS1 |
| TUBE1 | ZFPM2-AS1 |
| GABPB1 | ZFPM2-AS1 |
| PIK3CA | ZFPM2-AS1 |
| ATM | ZFPM2-AS1 |
| BACH1 | ZFPM2-AS1 |
| RB1 | LINC00472 |
| ANGPTL7 | LINC00472 |
| KLHL24 | LINC00472 |
| TUBE1 | LINC00472 |
| IL33 | LINC00472 |
| MAP3K5 | LINC00472 |
| IREB2 | LINC00472 |
| SP1 | LINC00472 |
| GABPB1 | LINC00472 |
| PIK3CA | LINC00472 |
| ZEB1 | LINC00472 |
| MAPK8 | LINC00472 |
| PRKAA1 | LINC00472 |
| TGFBR1 | LINC00472 |
| ANO6 | LINC00472 |
| ATM | LINC00472 |
| FBXW7 | LINC00472 |
| BACH1 | LINC00472 |
| ARNTL | MBNL1-AS1 |
| ANGPTL7 | MBNL1-AS1 |
| KLHL24 | MBNL1-AS1 |
| TUBE1 | MBNL1-AS1 |
| IREB2 | MBNL1-AS1 |
| GABPB1 | MBNL1-AS1 |
| PIK3CA | MBNL1-AS1 |
| ZEB1 | MBNL1-AS1 |
| MAPK8 | MBNL1-AS1 |
| ATM | MBNL1-AS1 |
| FBXW7 | MBNL1-AS1 |
| BACH1 | MBNL1-AS1 |

MRGs

| METTL14 | MBNL1-AS1 |
| --- | --- |
| RBM15 | MBNL1-AS1 |
| YTHDC2 | MBNL1-AS1 |
| FMR1 | MBNL1-AS1 |
| METTL14 | LINC00472 |
| RBM15 | LINC00472 |
| YTHDC2 | LINC00472 |
| FMR1 | LINC00472 |
| RBM15 | ZFPM2-AS1 |
| HNRNPA2B1 | TMPO-AS1 |
| RBM15 | LINC01572 |
| YTHDC2 | LINC01572 |
| YTHDF3 | LINC01572 |
| FMR1 | LINC01572 |
